# Assessing Agreement between Multiple Raters with Missing Rating Information, Applied to Breast Cancer Tumour Grading

**DOI:** 10.1371/journal.pone.0002925

**Published:** 2008-08-13

**Authors:** Thomas R. Fanshawe, Andrew G. Lynch, Ian O. Ellis, Andrew R. Green, Rudolf Hanka

**Affiliations:** 1 Department of Medicine, Lancaster University, Lancaster, United Kingdom; 2 Department of Oncology, Li Ka Shing Centre, University of Cambridge, Cambridge Research Institute, Cambridge, United Kingdom; 3 University of Nottingham, Nottingham, United Kingdom; 4 Division of Pathology, School of Molecular Medical Sciences, University of Nottingham, Nottingham, United Kingdom; 5 Wolfson College, University of Cambridge, Cambridge, United Kingdom; University of East Piedmont, Italy

## Abstract

**Background:**

We consider the problem of assessing inter-rater agreement when there are missing data and a large number of raters. Previous studies have shown only ‘moderate’ agreement between pathologists in grading breast cancer tumour specimens. We analyse a large but incomplete data-set consisting of 24177 grades, on a discrete 1–3 scale, provided by 732 pathologists for 52 samples.

**Methodology/Principal Findings:**

We review existing methods for analysing inter-rater agreement for multiple raters and demonstrate two further methods. Firstly, we examine a simple non-chance-corrected agreement score based on the observed proportion of agreements with the consensus for each sample, which makes no allowance for missing data. Secondly, treating grades as lying on a continuous scale representing tumour severity, we use a Bayesian latent trait method to model cumulative probabilities of assigning grade values as functions of the severity and clarity of the tumour and of rater-specific parameters representing boundaries between grades 1–2 and 2–3. We simulate from the fitted model to estimate, for each rater, the probability of agreement with the majority. Both methods suggest that there are differences between raters in terms of rating behaviour, most often caused by consistent over- or under-estimation of the grade boundaries, and also considerable variability in the distribution of grades assigned to many individual samples. The Bayesian model addresses the tendency of the agreement score to be biased upwards for raters who, by chance, see a relatively ‘easy’ set of samples.

**Conclusions/Significance:**

Latent trait models can be adapted to provide novel information about the nature of inter-rater agreement when the number of raters is large and there are missing data. In this large study there is substantial variability between pathologists and uncertainty in the identity of the ‘true’ grade of many of the breast cancer tumours, a fact often ignored in clinical studies.

## Introduction

### Background

The problem of assessing agreement between two or more assessors, or raters, is ubiquitous in medical research. Some of the many examples can be found in the fields of radiology, epidemiology, diagnostic medicine and oncology [1].

The problem can be split into two broad categories, according to the presence or absence of a ‘gold standard’, defined as an infallible method for determining the quantity of interest [2]. One might further subdivide these two cases according to whether the quantity of interest is categorical (such as the presence of absence of a disease) or continuous (such as a measurement of blood glucose levels). Ordinal quantities (such as ultrasound score, measured on a 1–5 scale) can be treated either as a separate category, or analysed as if either categorical or continuous. The case in which a gold standard is available has been extensively studied, and appropriate statistical methods have been developed. Often, useful summary statistics such as sensitivity and specificity, positive and negative predictive values and positive and negative likelihood ratios are calculated to assess the adequacy of a diagnostic test [2], [3].

In this paper we look at a particular case of the second category, in which a gold standard measure is not available. Uebersax and Grove [4] define three basic designs used for the analysis of inter-rater agreement data of this type:

1. The fixed panel design, in which each sample is rated by each rater.
2. The varying panel design, in which each sample is rated by a different set of raters. Raters are ‘anonymous’, in the sense that while it might be possible for a single rater to rate more than one sample, this event would either be unrecorded or not considered in the analysis.
3. The replicate measurement design, in which samples are rated on multiple occasions by each rater.

In such examples the calculation of simple summary statistics such as sensitivity and specificity is not possible, but there is a large literature on alternative measures such as the Kappa coefficient [5], whose merits have been debated at length. Although multi-rater versions of the Kappa coefficient exist, their use is not uncontroversial and they rely on a design in which each rater provides a rating for each sample [6]. Alternative methods focus on modelling patterns of agreement, and log-linear models [7] and latent trait and latent class models [8] have been widely used for this purpose.

We begin by reviewing the existing methods that have been used to assess inter-rater reliability for ordinal or categorical outcome variables in which there is no gold standard measure. We then develop a new, intuitive summary statistic for a motivating example, consisting of grading breast cancer tumour samples, in which the number of raters is large, and there is missing rating information (i.e. a rating is not available from each rater for each sample), our overall aim being to summarise the extent to which individual raters agree with the group of raters as a whole. We assess the suitability of this simple measure by comparing results with those from a Bayesian latent trait model for an ordered categorical response, and conclude by summarising the usefulness of the two methods in the analysis of this particular type of agreement data.

### Motivating Example

Breast cancer is a heterogenous disease and is highly variable in shape, size and character. However, a substantial amount of useful prognostic information is available from the careful histopathological examination of routine breast carcinoma specimens [9]. One of the most fundamental aspects of oncological pathology, which has undoubtedly stood the test of time, has been the recognition that the detailed morphological structure of tumours, i.e. histological grade, is strongly related to their degree of malignancy. In 1928, Patey and Scarff determined that only three factors – tubule formation, nuclear pleomorphism and hyperchromatism – were of importance in breast cancer grading [10]. Their method has formed the basis of all subsequent grading systems.

The Nottingham method, outlined in Table 1, is the most widely used method and overall grade is assigned as follows: Grade 1 - well differentiated - 3–5 points, Grade 2 - moderately differentiated - 6–7 points, Grade 3 - poorly differentiated - 8–9 points. It has been validated through long-term follow up of over 3000 patients confirming conclusively the highly significant relationship between histological grade and prognosis; survival worsens with increasing grade [11]. The method has now been adopted for use in the pathological data-set of the United Kingdom National Health Service Breast Screening Programme [12] and in the USA and Europe.

**Table 1:** Summary of the semi-quantitative method for assessing histological grade in breast carcinoma.

Feature	Score
**Tubule formation**	
Majority of tumour (>75%)	1
Moderate degree (10–75%)	2
Little or none (<10%)	3
**Nuclear pleomorphism**	
Small, regular uniform cells	1
Moderate increase in size and variability	2
Marked variation	3
**Mitotic counts**	
Dependent on microscope field area	1–3

The perceived poor reproducibility and consistency of grading systems has been improved by use of semi-objective scoring systems and adherence to written criteria such as those provided by the Nottingham method [13], [14], [15], but these studies have highlighted the need for grading to be carried out by trained histopathologists who work to an agreed protocol. A number of previous authors have found ‘moderate’ agreement between pathologists in this regard [16], [17], [18], and these conclusions are typically based on studies that use a small number of pathologists and simple methods of statistical analysis such as the Kappa coefficient. We aim to test these findings using a much larger data-set than those previously reported in the published literature.

Our data-set consists of grades provided by 732 pathologists (hereafter termed ‘raters’) for histological tissue sections from 52 breast cancer tumour samples (hereafter termed ‘samples’) circulated between 2001 and 2004, in eight twice-yearly batches. Not every rater was sent all of the samples, but raters gave grades to an average of 33 of the 52 samples (range 2 to 52 samples, interquartile range 20 to 47 samples). In the terminology of Uebersax and Grove [4], our example provides a variation on the varying panel design. Samples are rated by different sets of raters, assumed to have been chosen at random so as to be representative of the underlying population of raters, but we term the raters as ‘onymous’, in the sense that which ratings belonged to which rater can be identified in all of the samples (although in our example the identity of the raters is not disclosed).

1367 of the 25544 individual samples submitted to raters for grading (9%) were returned either ungraded or as ‘not assessable’. These instances have been removed from the data-set and are therefore treated as missing data in the same manner as samples that were not sent to raters. Each sample was graded by between 390 (53%) and 513 (70%) raters, which leaves around 36% of all sample-rater pairs that were ungraded and that are regarded as missing data. The primary aims of the project are to provide information concerning the extent of inter-rater agreement in assigning grades to samples, and to ascertain whether there is any evidence that some raters consistently give values different to the majority. This might be the case if, for example, raters were to interpret aspects of the grading scale and guidelines in different ways.

Observed marginal data from an illustrative selection of samples and raters are shown in the first five columns of Tables 2 and 3 respectively. Table 3 gives some indication of the extent of the variability between raters in the distribution of grades that they assign, some raters appearing, superficially, to have a greater tendency to give high grades than others.

**Table 2:** The distribution of grades assigned to a subset of tumour samples.

Sample	Observed : n (%)				Simulated : %			Estimated	
	G1	G2	G3	Ungraded	G1	G2	G3	μ_i_ (s.e)	λ_i_ (s.e)
1	386 (93.2)	28 (6.8)	0 (0)	318	93.0	6.7	0.2	−5.1 (0.6)	1.0 (0.2)
6	326 (70.1)	137 (29.5)	2 (0.4)	267	69.8	29.2	1.0	−3.0 (0.1)	1.0 (0.1)
52	223 (43.4)	285 (55.6)	5 (1.0)	219	43.2	56.0	0.8	−1.8 (0.1)	1.5 (0.1)
39	183 (39.3)	258 (55.3)	25 (5.4)	266	38.6	56.1	5.2	−1.4 (0.1)	0.9 (0.1)
18	46 (10.1)	393 (86.1)	17 (3.7)	276	10.4	85.5	4.1	−0.3 (0.1)	1.8 (0.1)
46	77 (15.6)	349 (70.6)	68 (13.8)	238	16.0	70.2	13.9	−0.1 (0.1)	1.0 (0.1)
43	23 (4.8)	376 (78.3)	81 (16.9)	252	5.5	77.4	17.1	0.6 (0.1)	1.3 (0.1)
48	6 (1.2)	209 (42.1)	282 (56.7)	235	1.2	41.6	57.2	2.3 (0.1)	1.1 (0.1)
8	1 (0.2)	161 (34.4)	306 (65.4)	264	0.6	33.6	65.8	2.7 (0.1)	1.2 (0.1)
13	0 (0)	4 (0.9)	454 (99.1)	274	0	1.0	99.0	6.7 (1.5)	1.2 (0.4)

Grades (G1–G3) assigned to a selection of ten breast tumour samples by 732 pathologists, with simulated results and parameter estimates from the Bayesian latent trait model.

**Table 3:** The distribution of grades assigned by a subset of pathologists.

Rater	Observed : n (%)				Agreement Score	Simulated No. of samples in agreement with majority (s.d.)	Estimated	
	G1	G2	G3	Ungraded			b_12_ (s.e)	b_23_ (s.e)
156	20 (65)	8 (26)	3 (10)	21	0.41	27.9 (3.3)	0.8 (0.4)	5.1 (0.5)
273	22 (48)	11 (24)	13 (28)	6	0.64	39.2 (2.9)	−0.6 (0.3)	2.6 (0.5)
275	18 (40)	7 (16)	20 (44)	7	0.73	41.3 (2.7)	−1.4 (0.3)	1.1 (0.4)
137	20 (39)	13 (25)	18 (35)	1	0.76	41.7 (2.6)	−1.2 (0.3)	2.0 (0.4)
247	5 (11)	28 (62)	12 (27)	7	0.68	41.0 (2.4)	−3.5 (0.4)	2.9 (0.5)
500	14 (27)	21 (40)	17 (33)	0	0.76	43.2 (2.5)	−2.1 (0.4)	2.2 (0.4)
335	7 (23)	10 (33)	13 (43)	22	0.72	42.7 (2.6)	−2.0 (0.4)	1.8 (0.5)
617	13 (26)	13 (26)	24 (48)	2	0.73	41.5 (2.8)	−2.3 (0.4)	0.8 (0.4)
521	1 (6)	4 (25)	11 (69)	36	0.65	38.8 (3.6)	−3.3 (0.7)	0.5 (0.6)
143	0 (0)	11 (55)	9 (45)	32	0.50	35.7 (3.3)	−5.0 (0.6)	0.4 (0.5)

Grades (G1–G3) assigned by a selection of ten pathologists to 52 breast cancer tumour samples, with estimated agreement scores, and simulated results and parameter estimates from the Bayesian latent trait model.

## Methods

In this section we discuss existing methods for analysing inter-rater agreement data, and describe two methods that we use to analyse the breast cancer tumour data.

### Existing methods

#### The Kappa coefficient

One summary statistic, the roots of which are found in the psychology literature, is particularly commonly used in papers reporting inter-rater agreement with a categorical outcome: the Kappa coefficient [5].

The rationale for the Kappa coefficient and other similar measures of agreement is that they are chance-corrected, in the sense that they attempt to allow for the fact that for discrete or ordinal outcomes there will be a non-zero probability *p_e_* that two raters will agree on a sample simply by guessing, thus making the observed probability of agreement *p_o_* appear artificially high.

Given *n* pairs of ratings, an estimate of the true Kappa coefficient *κ* is given by the expression
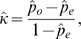
with approximate standard error
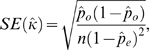
where *pˆ* *_o_* and *pˆ* *_e_* are estimates of the respective probabilities and *n* is the number of samples. Further details of the computation of the Kappa coefficient are given by Siegel and Castellan [19].

There are a large number of papers both advocating and criticising the use of the Kappa coefficient for assessing inter-rater agreement. Briefly, the main criticisms are that its interpretation is often based on somewhat arbitrary guideline values, leading to problems of interpretation; that it is heavily dependent on observed marginal proportions and thus the case-mix of the samples used; that it can be severely misleading in degenerate cases in which one or more of the outcome categories is uncommon; and that it lacks natural extensions when there is more than one outcome of interest or when multiple raters are used [6], [20], [21], [22]. Other chance-corrected measures have also come in for criticism [23]. Weighted versions of the Kappa coefficient exist for the case of multiple, ordered categories [24], [25], but interpretation is clouded further by an often arbitrary choice of weights for each category. In the context of the breast cancer tumour data, there are additional complications: there is a large number of raters, and an onymous varying panel design, whereas the Kappa coefficient requires a fixed panel design.

#### Latent trait and latent class modelling

Latent trait and latent class modelling have become increasingly popular in recent years for analysing inter-rater agreement data. Summaries are provided by Langeheine and Rost [8] and a recent review journal edition [26]. In the latent trait model, it is assumed that there exists an unobserved, or latent, continuous variable that represents key properties of each sample being rated. In the classical framework, a distributional form of latent trait levels is assumed for each group of samples (for example, for tumour samples with true grades 1, 2 and 3, or for disease cases and controls). Typically, both the parameters that characterise these distributions, and thresholds by which raters transform the latent variables into observed ratings, are estimated by maximum likelihood [4]. We discuss further details of the latent trait model later.

In latent class modelling, samples are regarded as belonging to exactly one of *c* unobserved categories, and conditional probabilities of a sample being assigned each particular rating value, given its latent class, are estimated. Often the appropriate choice of *c* is unknown in advance, and is estimated, or models with different values of *c* are compared [27], [28].

Although latent trait models have received some criticism because the underlying trait variable lies on an arbitrary, uninterpretable scale [1], other authors have shown how estimated parameters are related to familiar summary statistics such as sensitivity, specificity and predictive values [27], [29]. Models of this type have consequently been used in a number of different applications, including inter-rater agreement [30], [31].

#### Other methods

One major class of models that has been used for agreement data is that of log-linear models for categorical data, as described by Agresti [7]. Originally developed from quasi-symmetry models for pairs of raters, these models have been adapted to produce a global measure of agreement for multiple raters [32]. Typically such models provide a means of assessing departure of observed data from the diagonals of either multiple two-dimensional contingency tables, or a single high-dimensional table, with parameters estimated directly by maximisation of the likelihood function. While feasible for small numbers of raters, this procedure quickly becomes computationally prohibitive as the number of raters increases - for example, the exceedingly sparse single contingency table representing the breast cancer tumour data would have dimension 3^732^.

Other summary statistics that have been proposed include Yule's *Y*, the odds ratio and the Phi coefficient, whose relative merits are discussed by Feinstein and Cicchetti [6], and Cicchetti and Feinstein [22]. Martin Andres and Femia Marzo suggest an alternative chance-corrected coefficient, Delta [33]. These methods all require a fixed panel design. Landis and Koch [34] propose a method based on variance partitioning in which agreement is summarised using intra- and inter-rater correlation coefficients. For the varying panel design, James [35] suggests an ‘impartiality index’ as a means to identify categories in which there occur a higher proportion of disagreements than expected given the marginal proportions for each category. Altaye et al. [36] give maximum likelihood estimates for relevant parameters in the fixed panel multi-rater agreement problem, although the computation of these maximum likelihood estimates is infeasible if the number of raters is large or if rating data are sparse. Nelson and Pepe [1] provide a novel graphical display as a means for preliminary analysis for fixed panel data (a three-dimensional plot illustrating how the marginal proportions of the response categories vary according to the average response category across all raters). A potential limitation of this method is the clear dependence between the plotted quantities, and the consequent difficulty in interpretation.

### Proposed methods

We use the breast cancer tumour data to demonstrate and compare two methods for analysing agreement data with a large number of raters and an onymous varying panel design. Our proposed methods are designed to reflect the extent to which the distribution of ratings provided by individual raters agrees with that provided by all raters.

#### The agreement score

An easily-computed, intuitive summary statistic is a simple agreement score *s_j_*, which can be calculated for each rater *j* and which is based on the marginal distribution of grades given to each sample.

Let *g_ij_* be the observed grade assigned to sample *i* by rater *j*, *N_j_* be the number of samples given a rating by rater *j*, and *n_i,g_* be the observed number of raters giving grade *g* to sample *i*, for *g* = 1,2,3 and *i* = 1,…,*m*, where *m* = 52 in the example. Then the contribution of sample *i* to the agreement score of rater *j* (*j* = 1,…,732), is
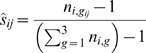
(1)The contribution is zero if *j* does not give a rating to *i*, and the agreement score of *j* is estimated as 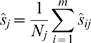
(2)Our initial assumptions are that different samples are independent and that in the case of incomplete rating data no information can be gleaned from the pattern of missing data (i.e. that there is no preferential selection of which samples are allocated to or returned by the various raters). In the special case that each rater gives a rating to each sample (the fixed panel design) and the further null assumption that all *R* raters are equally proficient, we can regard the distribution of the total number of raters, say (*Y*_1_,*Y*_2_,*Y*_3_), assigning grades 1, 2 and 3 respectively to a given sample *i* as a realisation of a Multinomial (*R*; *p_i_*_,1_, *p_i_*_,2_, *p_i_*_,3_) random variable, where *p_i,g_* denotes the probability of a randomly-chosen rater giving grade *g* to sample *i*.

Then for any rater *j*, we have from standard properties of the multinomial distribution that
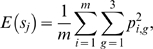
(3)and
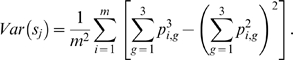
(4)
*s_j_* can be regarded as an estimator of the overall proportion of raters that will agree with a given rater *j* on the grade of a randomly-chosen sample. Possible values of *s_j_* therefore range from 0 to 1, with a value of 1 indicating that there was unilateral agreement on grade for every sample, and a value of 0 indicating that a particular rater did not give the same grade as any other rater for any sample. Under the null hypothesis, the minimum possible value of *E*(*s_j_*) is 1/3, occurring only in the highly unlikely case that *p_i_*_,1_ = *p_i_*_,2_ = *p_i_*_,3_ = 1/3 for each sample *i*. Note that the agreement score depends on the nature of the tumour samples being rated, so, as for most studies of inter-rater agreement, comparison between studies with differing case-mixes of tumour samples requires care.

Importantly, neither the mean nor the variance of the agreement score depends on the number of raters who rate each sample, which enables a fair comparison of agreement scores between raters to be made in the presence of incomplete rating data. In practice the *p_i,g_* in (3) and (4) will be unknown, and will be replaced by maximum likelihood estimates. It should also be noted that, for two different raters *j* and *k*, agreement scores *s_j_* and *s_k_* are not independent: there is a small positive covariance between *s_j_* and *s_k_* that has a negligible impact for large sample sizes. From (4), the sampling variance of the agreement score of a given rater decreases with the number of samples graded by the rater, a key factor in the interpretation of the agreement score. In order to assess the level of evidence for the hypothesis that not all raters interpret the grading scale in the same way, we consider a graph similar to a funnel plot [37], in which we add upper and lower confidence ‘envelopes’ calculated by simulation under the null assumption that *p_i_*_,1_ = *p*_1_, *p_i_*_,2_ = *p*_2_ and *p_i_*_,3_ = *p*_3_ for each rater *i*. We use the observed marginal proportions of grades 1, 2 and 3 for each sample *g* as plug-in estimates of the true population proportions *p*_1,*g*_, *p*_2,*g*_ and *p*_3,*g*_. The steps required to create such a plot are:

1. Fix *h*≤*m*, the number of samples graded by a hypothetical rater.
2. Select *h* of the 52 samples at random, say *y*_1_,…,*y_h_*.
3. For samples *y*_1_,…,*y_h_*, simulate grades *g*_1_,…,*g_h_* from the observed empirical distributions of grades given to the sample (i.e. for a given sample, with probability of selecting grade *j* proportional to the proportion of raters who assigned grade *j* to the sample).
4. Estimate the agreement score based on the simulated grades *g*_1_,…,*g_h_* using (1) and (2).
5. Repeat steps 2–4.

We can then estimate the distribution function of the agreement score based on a large number of replications for each *h*. The upper and lower confidence envelopes can be added to a plot of agreement score against number of samples graded, and used to indicate raters who behave anomalously compared with the majority. While we draw an analogy between the resulting plot and the funnel plot used in other contexts, the two differ in the sense that there is no pre-defined tolerance limit to which we compare scores of individual pathologists: such a limit would depend on the case-mix of tumour samples used. Also, note that the envelopes are not independent of the observed data, but are intended simply to give a visual indication of how the variability of the agreement score changes with the number of samples rated.

#### Bayesian latent trait model

Using a Bayesian formulation of the problem enables relevant parameters to be estimated without recourse to maximising the likelihood function directly [4], which would be impractical for our application given the large number of raters and incomplete data structure.

We think of the categorical response variable as representing an underlying, latent, scale (c.f. the ‘Bones’ example in [38]) indicative of the severity of the tumour. We regard the act of a rater grading a sample as estimating its true severity as a number on the latent scale, and comparing this position with two grade boundaries that ‘separate’ grades 1 and 2, and 2 and 3 respectively. These grade boundaries are allowed to vary between raters. If rater *j* estimates the position of a given sample on the latent scale as *x_j_*, then he will assign grade 1 if *x_j_*<*b*_12,*j*_, grade 2 if *b*_12,*j*_<*x_j_*<*b*_23,*j*_ and grade 3 if *x_j_*>*b*_23,*j*_, where *b*_12,*j*_ and *b*_23,*j*_ are, respectively, the lower and upper grade boundaries on the latent scale according to its interpretation by rater *j*.

This can be represented by a cumulative logit model of the form
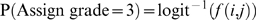


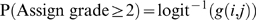
for suitably-chosen functions *f* and *g*. We choose the following forms for *f* and *g*:
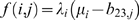


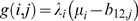
Our choice of these functional forms is motivated by the fact that the parameters are easily interpretable: *μ_i_* can be thought of as the latent measure of the severity of sample *i*, *λ_i_* as the clarity of the sample (i.e. the ease with which it can be assessed), and *b*_12,*j*_ and *b*_23,*j*_ as the grade boundaries of rater *j* as described above.

We choose priors for the parameters as follows. We give the average of the two boundaries *b*_12_ and *b*_23_, *b_av_* say, a Normal prior with zero mean, and a hyperparameter for the inverse of the variance that itself has a Normal(0,1) distribution, truncated to be positive. We give half the distance between the two grade boundaries, 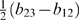, say, a Normal prior with a mean of two, truncated to be positive to constrain the *b*_12_ boundary to lie below the *b*_23_ boundary. The inverse of the variance of this prior is again a hyperparameter with a Normal(0,1) distribution truncated to be positive.

We give the tumour severity parameters *μ* a Normal prior, with both mean and variance set to be hyperparameters. We also give the hyperparameter for the inverse of the variance a Normal(0,1) distribution truncated to be positive, and that for the mean a Normal(0,4) distribution. We assign to the tumour clarity parameters *λ* a truncated Normal prior with mean zero and an inverse-variance hyperparameter that has a Normal(0,1) distribution truncated to be positive.

In order for the model to be fitted, certain conditions must hold. Consider a bipartite graph with nodes representing samples and raters, in which edges connect raters to the samples they saw. The graph must be connected in order for the parameters to be identifiable and to enable reasonable comparison between the grade boundaries of different raters. The graph for this data-set is 2-connected, thus ensuring parameter identifiability.

Finally, we can obtain new, simulated, sets of rater/tumour observations by repeatedly sampling from the fitted Bayesian model. For each set of simulations, we record the number of raters assigning grades 1, 2 and 3 to each tumour and the majority grade for each tumour. Using data from 1250 simulations, we estimate the probability that a given rater would agree with the majority for a given tumour for each rater/tumour pair. The simulated data allow the estimation of two marginal probabilities, *q_j_* and *r_i_* say, the first giving a measure of the performance of each rater *j* (i.e. the probability of the rater being in the majority for a sample chosen at random) and the second giving a measure of the difficulty associated with grading each sample *i* (i.e. the proportion of raters giving the consensus grade for the sample).

**Figure 1:**
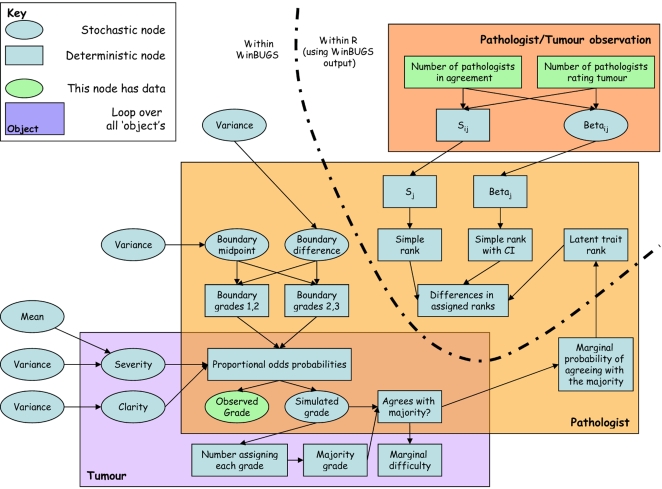
Schematic representation of the Bayesian latent trait model.

The model was fitted in WinBUGS 1.4.2 [39], and other calculations were performed using R version 2.5 [40]. Code used for the analysis is available in supplementary Statistical analysis file S1, S2, S3, S4, S5, S6, S7 and S8 at the journal website. A schematic representation of the model used is shown in Figure 1.

## Results

### The agreement score

Calculated values of the agreement score amongst the 732 raters range from 0.35 to 0.87 (mean 0.72). The scores from ten raters are shown in Table 3. The theoretical mean agreement score given the marginal proportions for each grade for each sample using (3) is 0.73.

**Figure 2:**
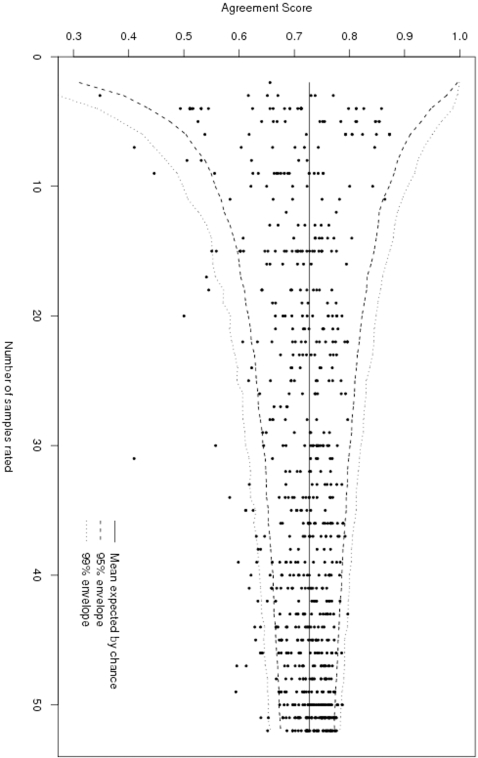
Estimated agreement score, with 95% and 99% confidence envelopes, for 732 pathologists. Plot of estimated agreement score against number of samples rated, with confidence envelopes within which 95% and 99% of raters would be expected to lie if all raters were equally proficient.

Figure 2 shows the funnel-type plot of the agreement scores for all raters, with 95% and 99% confidence envelopes based on 10000 replications and the median simulated agreement score added. 61 raters (8.3%) lie outside the 95% envelope, and 31 raters (4.2%) outside the 99% envelope, suggesting substantial differences between raters in the way by which grades are assigned. Note that of the raters lying outside the 99% envelope, almost all lie below the lower bound, rather than above the upper bound. We would not normally encourage using the funnel-type plot as a means of picking out individuals whose discrepancies may be attributable to chance alone, but in this example one point lies so much further from the envelope than the rest that the corresponding rater warrants further investigation: rater 156, who rated 31 samples and whose agreement score is just 0.41. We can see from Table 4 that this particular rater has a marked tendency to underestimate grades compared to the consensus value (the observed modal grade amongst all raters).

**Table 4:** Examination of grades assigned by a single pathologist.

Rating of rater 156	Consensus grade		
	G1	G2	G3
G1	8	12	0
G2	0	1	7
G3	0	0	3

Comparison of the 31 breast cancer tumour sample grades (G1–G3) given by the single rater 156 with the consensus grade.

### Bayesian latent trait model

Tables 2 and 3 contain parameter estimates and results of the simulations from the fitted model. From simulation, the mean proportion assigning each grade to a given sample was very similar to the observed proportion of grades (Table 2).

**Figure 3:**
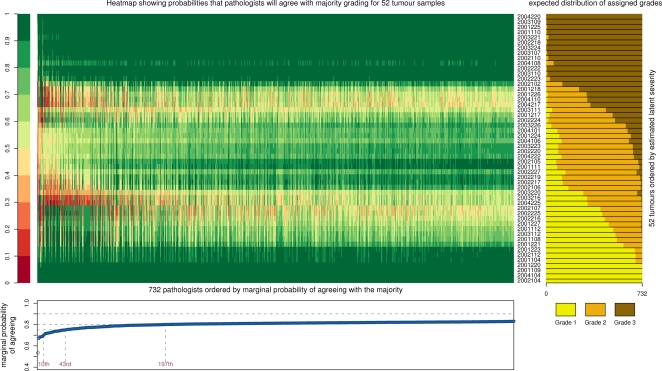
Summary of results of the Bayesian latent trait model. The main body of the plot is a heatmap showing the probability that raters (columns) agree with the consensus grade for each sample (rows). Raters are ordered in terms of estimated probability of agreeing with the majority, and samples in terms of estimated latent severity. The right-hand panel shows the expected distribution of assigned grades for each sample, and the bottom panel shows, for each rater, the marginal probability of agreeing with the consensus grade.

The simulation results are summarised in Figure 3, in which the estimated probabilities of each rater agreeing with each sample's modal grade are plotted. Red values indicate low probabilities and green values high probabilities. The panel on the right-hand side of Figure 3 indicates the expected proportion of ratings assigned to each of the three grades. There are around 15 samples for which consensus on the modal grade is unclear, which naturally leads to relatively low probabilities of agreeing with the modal grade for most raters. At the other extreme, some samples were regarded almost equivocally as grades 1 or 3, while the consensus was weaker for the samples for which grade 2 was the modal value, a result also noted by other authors [18].

**Figure 4:**
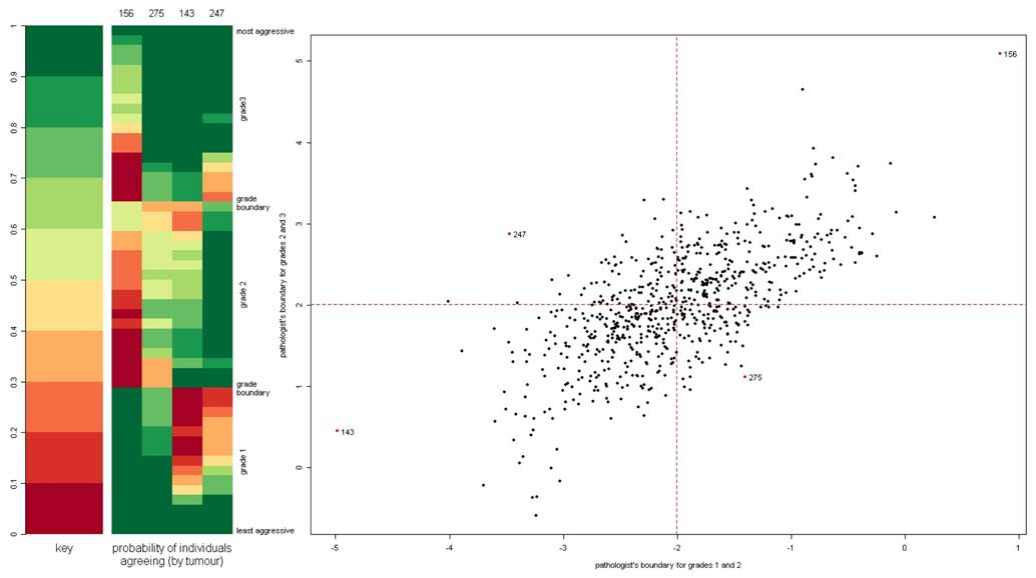
Comparison of grade boundaries, estimated from the Bayesian latent trait model, for 732 pathologists. Scatter-plot of estimated grade boundaries from the Bayesian latent trait model, in which the estimated grade boundary *b*_12,*j*_ is plotted against *b*_23,*j*_ for each rater *j*. The left-hand panel shows the estimated probability *q_j_* of agreeing with the majority for four raters, one from each quadrant of the graph, as indicated on the main plot.

Although the estimated values of the grade boundary parameters *b*_12,*j*_ and *b*_23,*j*_, shown for a selection of raters in Table 3, have no direct interpretation themselves, they can be plotted to compare rating patterns of the raters relative to one another (Figure 4). The four quadrants of the graph indicate four types of rater behaviour in tumour classification. The panel to the left of the graph shows in detail, for one rater in each quadrant, the estimated probability of the rater agreeing with the majority for each sample, where the samples have been ordered by their estimated severity. As already noted, rater 156 tends to under-estimate grades compared to the majority, while rater 143 tends to over-estimate. Rater 247, with a low 1–2 boundary and a high 2–3 boundary, tends to assign many grade 2s, while rater 275, with a high 1–2 boundary and a low 2–3 boundary, tends to assign few grade 2s. The elliptical nature of this plot, with a preponderance of points in the first and third quadrants, suggests that relative over- or under-estimation of the grade boundaries are the most common patterns that leads to disagreement between raters. We note that for computational ease we implemented the model in a manner that will impart some small correlation on the relationship between *b*_12,*j*_ and *b*_23,*j*_, but simulations suggest that this will not be responsible for the magnitude of correlation seen here. As indicated by Table 3, the expected number of samples for which a rater agrees with a consensus changes substantially only when a rater's rating behaviour is extremely atypical.

**Figure 5:**
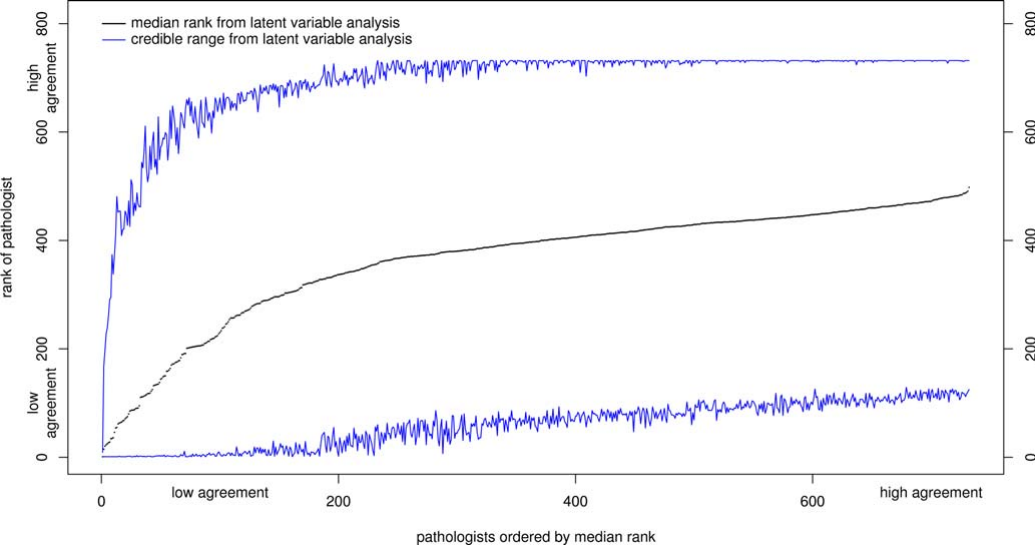
Estimated ranks of 732 pathologists. Plot of the raters' ranks, estimated from the Bayesian latent trait model, with 95% credible limits.

Figure 5 shows estimated ranks of each rater based on estimated *q_j_* values from 625 simulations from the fitted model, plotted with 95% confidence intervals and ordered by the point estimate of the rank. The overall impression is of very wide intervals, encompassing the majority of the range of ranks, for all but the lowest-ranked few raters. The wide intervals are a consequence of both the small number of samples seen by many raters and the well-known difficulty of estimating ranks precisely.

### Comparison of the methods

**Figure 6:**
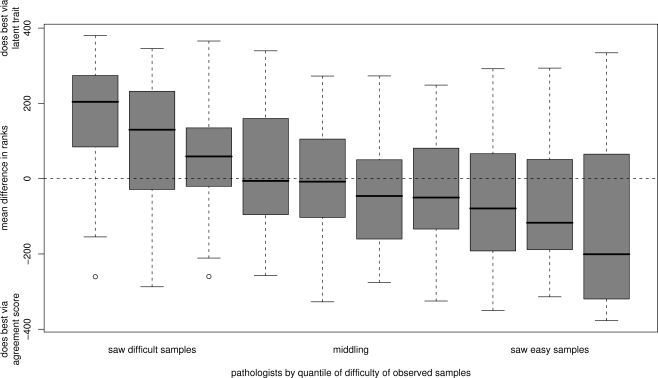
Comparison of the two analysis methods. Plot of differences in raters' estimated ranks for the two methods against a measure of the difficulty of the samples seen by each rater, grouped by deciles. This measure of difficulty is calculated as the average, taken over the samples seen by each rater, of the estimated values of *r_i_*.

Both the agreement score method and the Bayesian latent trait model indicate heterogeneity between raters for our data-set. We hypothesised that the agreement score method might give an unduly optimistic assessment of rater performance for raters who had seen a subset of samples that were relatively easy to grade. Therefore, in order to compare the two methods of analysis, we consider the estimated difference in raters' ranks from the two methods. We plot this against the estimate, averaged over the samples seen by each rater, of the *r_i_* (Figure 6). The clear trend verifies the anticipated result: raters who by chance saw ‘easy’ samples tend to have more favourable ranks by the agreement score method, which is rectified by the latent trait model.

## Discussion

We have developed and compared two methods for inter-rater agreement analysis of data in which there is no gold standard, a large number of onymous raters, and incomplete rating information.

The agreement score is a simple, non-chance-corrected statistic that can be easily calculated and potentially used in order to provide some evidence whether there may be raters whose behaviour is discrepant compared to that of the majority. It can therefore be regarded as a measure of the relative agreement between an individual rater and a population of raters. For our data, we found strong evidence that there were certain raters whose levels of agreement with the majority were worse than would be expected by chance. There was no evidence that there were raters who were better than chance, perhaps unsurprisingly - it is easier to envisage reasons why a single rater might record an unusually low score than an unusually high one.

Although the agreement score is dependent on the case-mix of the samples used in a particular study, it has a straightforward interpretation as the probability that a given rater will agree with another randomly-chosen rater on a randomly-chosen sample, and can be displayed graphically in a way that avoids misleading rankings. This may be a useful tool in the preliminary analysis of data of this type, and can be used to identify potentially discrepant raters as a first step in determining possible reasons why they may differ from the majority.

We have demonstrated, using the Bayesian latent trait model, a way by which to estimate both the performance of raters, via estimated grade boundary parameters, and the marginal distributions of ratings given to each sample. The agreement score for particular raters may be misleading for raters who, by chance or otherwise, have only rated a selection of samples that are unusually easy or difficult to classify. In particular, we have shown that raters who rated ‘easy’ samples tend to have unjustifiably high values of the agreement score. We therefore believe that the latent trait method is of particular value if there is missing rating information and the number of raters is large, in which case the probability that some raters will see an unusually difficult or easy set of samples is increased.

In future work the method might also be developed to relax the assumption that missing rating information is uninformative, i.e. to test whether there was any preference on the part of the raters over which samples they chose to rate. For our example, this might occur if the pattern of missing data were related to the grade. The 9% of samples that were not rated most often occurred in groups of consecutively-numbered samples in single batches sent to certain individual raters, which in our opinion suggests that deliberate preferential rating is unlikely. In other extensions of the work, the method might be adapted for use with multivariate outcomes (e.g. to analyse the three components that constitute the grade), for ongoing rater assessment, and to deal with changes in rater behaviour or agreement over time (e.g. rater learning, or to check the impact of new grading guidelines). We do not anticipate that our proposed methods, designed for the case in which the number of raters is large, will be useful or even viable in small studies: much previous work has focussed on methods of analysis with fewer than ten raters (e.g. [3]). However, the precise extent to which the preferred methodology depends on the number of contributing raters also remains an open question.

Latent trait models have attracted some criticism because of the lack of interpretability of model parameters, owing to the arbitrary choice of latent scale [1]. However, by simulating from the posterior distributions of all parameters it is possible to provide estimates of directly interpretables1 quantities such as the probability that a rater will agree with the modal class. The method illuminates features of the raters' patterns of behaviour that would have not become apparent from a single-number summary such as the agreement score, a point that has also been noted with regard to the Kappa coefficient [32]. In the example considered in this paper, both the number of raters and the proportion of missing data were large, and therefore to find a model that could be fitted within the limits of computational feasibility was an important consideration.

Simulation enables ranks of raters, with plausible confidence limits, to be estimated, which could in principle be reported back to individuals. The wide confidence limits in our example, however, are illustrative of the great difficulty involved in estimating ranks precisely. Even with greater precision the practical value of knowing one's rank would be limited. Conversely, knowledge of the location of one's grade boundaries relative to other pathologists would be of potential interest and these measures require much less computation to obtain estimates than do the other results.

In the context of breast cancer tumour grading, our data show substantial variation between individual pathologists in the way in which grades are assigned to samples. This finding is broadly consistent with the existing literature: for example, Meyer et al. suggest that this is because ‘the level of agreement achievable is limited by the subjectivity of grading criteria’ [17]. This may have implications for clinical studies that treat grade as known on the basis of information given by just one or two raters - in many such cases the uncertainty associated with the ‘true’ grade may be too large to be overlooked. In summary, our modelling approach leads to richer conclusions than simple summary statistics can provide: for example, the most frequent source of discrepancies between raters appears to be due to consistent over- or under-estimation of the grade boundaries.

## Supporting Information

Statistical analysis file S1 Code used in the statistical analysis
(0.02 MB TXT)

Statistical analysis file S2 Code used in the statistical analysis
(0.01 MB TXT)

Statistical analysis file S3 Code used in the statistical analysis
(0.01 MB TXT)

Statistical analysis file S4 Code used in the statistical analysis
(0.01 MB TXT)

Statistical analysis file S5 Code used in the statistical analysis
(0.01 MB TXT)

Statistical analysis file S6 Code used in the statistical analysis
(0.01 MB TXT)

Statistical analysis file S7 Code used in the statistical analysis
(0.01 MB TXT)

Statistical analysis file S8 Code used in the statistical analysis
(0.01 MB TXT)
